# ATP stimulates chemokine production via a store-operated calcium entry pathway in C6 glioma cells

**DOI:** 10.1186/1471-2407-9-442

**Published:** 2009-12-15

**Authors:** Nattinee Jantaratnotai, Hyun B Choi, James G McLarnon

**Affiliations:** 1 Department of Anesthesiology, Pharmacology and Therapeutics, Faculty of Medicine, University of British Columbia, 2176 Health Sciences Mall, Vancouver, BC, V6T 1Z3, Canada; 2 Department of Pharmacology, Faculty of Science, Mahidol University, Rama VI Road, Phayathai, Bangkok 10400, Thailand

## Abstract

**Background:**

Glioma present as one of the most challenging cancers to treat, however, understanding of tumor cell biology is not well understood. Extracellular adenosine triphosphate (ATP) could serve as a critical signaling molecule regulating tumor development. This study has examined pharmacological modulation of calcium (Ca^2+^) entry through store-operated channels (SOC) on cellular expression and production of immune-cell mobilizing chemokines in ATP-stimulated C6 glioma cells.

**Methods:**

Calcium spectrofluorometry was carried out to measure mobilization of intracellular Ca^2+ ^[Ca^2+^]i following ATP stimulation of rat C6 glioma cells. Pretreatment with two inhibitors of SOC, SKF96365 or gadolinium, was used to examine for effects on [Ca^2+^]i. RT-PCR was performed to determine effects of purinergic stimulation on C6 cell expression of metabotropic P2Y receptors (P2YR) and the chemokines, monocyte chemoattractant protein-1 (MCP-1) and interleukin-8 (IL-8). ELISA was carried out to measure production of MCP-1 and IL-8 with ATP stimulation of glioma cells.

**Results:**

Application of ATP (at 100 μM) to C6 glioma induced an increase in [Ca^2+^]i with the response exhibiting two components of decay. In the presence of the SOC inhibitors, SKF96365 or gadolinium, or with Ca^2+^-free solution, ATP responses lacked a slow phase suggesting the secondary component was due to SOC-mediated influx of Ca^2+^. RT-PCR confirmed expression of purinergic P2Y-subtype receptors in C6 cells which would serve as a precursor to activation of SOC. In addition, ATP-stimulated C6 cells showed enhanced expression of the chemokines, MCP-1 and IL-8, with SKF96365 or gadolinium effective in reducing chemokine expression. Gadolinium treatment of ATP-stimulated C6 cells was also found to inhibit the production of MCP-1 and IL-8.

**Conclusion:**

These results suggest ATP-induced Ca^2+ ^entry, mediated by activation of SOC in C6 glioma, as a mechanism leading to increased cellular expression and release of chemokines. Elevated levels of MCP-1 and IL-8 are predicted to enhance the mobility of tumor cells and promote recruitment of microglia into developing tumors thereby supporting tumor growth.

## Background

Gliomas are a common form of human brain tumor but remain essentially incurable due to their innate characteristic of extreme invasiveness [1]. The development and progression of gliomas include reciprocal interactions between glioma cells with resident immune responding microglia and tumor-associated macrophages [2,3]. In particular, evidence suggests that tumor cells may produce mobilizing factors for microglia/macrophages and that chemokine responses of microglia could aid in establishing immunosuppressive environments facilitating tumor growth [4,5]. Among the most prominent glioma chemokine factors are monocyte chemoattractant protein-1 (MCP-1) and interleukin-8 (IL-8) which could induce recruitment of microglia/macrophage to help support tumor progression [6,7]. Moreover, MCP-1 has been shown to directly induce angiogenesis [8] while both chemokines also act as autocrine factors to drive the invasive phenotypes of the gliomas [6,9].

A spectrum of stimulatory signals is likely present in developing gliomas which activate microglial chemotactic responses and promote an overall immunosuppressive microenvironment. In particular, purinergic signaling pathways in glioma may be highly relevant in enhancement of chemotactic factors to recruit microglia to help sustain tumor growth [10,11]. Glioma cells both release and respond to ATP [12,13] with catabolism of ATP extremely low in glioma cells, compared to astrocytes, due to a marked reduction in expression and activity of enzymes that degrade ATP [14]. Importantly, depletion of ATP has been reported to reduce the size and invasive characteristics of tumors in an animal glioma model [15].

Evidence suggests that mobilization of intracellular calcium ([Ca^2+^]i), mediated by activation of the purinergic subtype receptor, P2X_7_R by the ligand 2',3'-(benzoyl-4-benzoyl)-ATP (BzATP), serves as a link between ATP stimulation of glioma and cellular expression of chemokine and cytokine factors [16]. However, roles of other purinergic family members were also suggested since antagonism of P2X_7_R had no effect to inhibit factor expression and only partially suppressed calcium responses [16]. In particular, it was speculated that subtype P2YR could also be activated by BzATP thereby increasing [Ca^2+^]i by a rapid release from endoplasmic stores followed by a subsequent sustained influx of the ion through store-operated channels (SOC). These findings suggested the relevance of study using the endogenous compound ATP as an activator of C6 cells to determine effects of pharmacological modulation of SOC-mediated Ca^2+ ^entry on cellular production of chemokines.

## Methods

### Materials

All chemicals were purchased from Sigma (St.Louise, MO) unless otherwise stated. Two inhibitors of SOC were employed in this work; gadolinium [17,18] and SKF96365 [19].

### Cell culture

C6 glioma cells were obtained from the American Type Culture Collection (ATCC, Manassas, VA). Cells from passage number 39-59 were used in this work. Cells were cultured in Kaighn's modification of Ham's F12 medium (F12K) with 2 mM l-glutamine modified by ATCC to contain 1.5 g/l sodium bicarbonate. The medium was then supplemented with 15% horse serum, 2.5% fetal bovine serum, 0.5 μg/ml fungizone (Invitrogen: GIBCO, Grand Island, NY) and 0.02 mg/ml gentamicin (Invitrogen: GIBCO). Cells were maintained in 100 mm culture dishes (SARSTEDT, Newton, NC) at 37°C in a humidified 5% CO_2 _air atmosphere.

### Calcium spectrofluorometry

The detailed procedure for calcium imaging was carried out as published [20]. Briefly, cultured C6 glioma cells were incubated with fura-2 acetoxymethyl ester (fura-2AM at 1 μM; Molecular Probes, Eugene, OR) and pluronic acid (at 1 μM) in normal physiological saline solution (PSS) for 20 min at room temperature (20-22°C). Cells were then washed with PSS solution containing (in mM): 126 NaCl, 5 KCl, 1 CaCl_2_, 1.2 MgCl_2_, 10 HEPES and 10 glucose (pH 7.4). In some experiments, Ca^2+^-free PSS was used; this solution had the same composition as PSS with the exception that EGTA was added (at 1 mM) with no CaCl_2_. Coverslips were placed in a perfusion chamber mounted on an inverted microscope (Zeiss, Jena, Germany) and fluorescence was measured through a 40× quartz objective lens. Alternating wavelengths (340/380 nm) of ultraviolet light were applied at 6-s intervals for excitation and fluorescence signals were measured at 510 nm of emission light. Signals were acquired from a digital camera (DVC-1310, DVC Co. Austin, TX) and were processed using an imaging system (Empix, Mississauga, ON, Canada) to determine ratios of the 340 and 380 nm intensities which were used as quantitative measures of fluorescence levels in this work.

### Semiquantitative RT-PCR

C6 glioma cells (8 × 10^5 ^cells/well) were plated in serum-free media and the experiment was carried out the following day with addition of ATP (100 μM) in the absence or presence of SKF96365 (25 μM) or gadolinium (1 μM) for 4 h. Total RNA was isolated using TRIzol (Invitrogen: GIBCO) and then processed for the first strand complementary DNA (cDNA) synthesis using Moloney murine leukemia virus (M-MLV) reverse transcriptase (Invitrogen: GIBCO). The cDNA products were then amplified by PCR using a GeneAmp thermal cycler (Applied Biosystems, Foster City, CA). Specific sense and antisense primers with the expected product sizes are shown in Table 1. PCR conditions were as follows: initial denaturation at 95°C for 6 min followed by a 25- to 35- cycle amplification program consisting of denaturation at 95°C for 45 s, annealing at 55-60°C for 1 min and extention at 72°C for 1 min. A final extention was carried out at 72°C for 10 min. For P2YR expression study, the PCR cycle was set at 35 cycles for all subtype receptors. Amplification of the constitutively expressed enzyme D-glyceraldehyde-3-phosphate dehydrogenase (GAPDH) was used as a reaction standard. The amplified PCR products were identified using 1.5% agarose gels containing ethidium bromide (final concentration 0.5 μg/ml) and visualized under ultraviolet light. The intensities of each band were measured by densitometry using NIH Image J 1.37b software (National Institute of Health, Bethesda, MD) and expressed as relative mRNA levels (mRNA levels normalized to GAPDH).

**Table 1:** Primer sequences and product sizes

P2Y_1_R sense	5'-TGGCGTGGTGTACCCTCTCAAGTC-3'	558 bp
P2Y_1_R antisense	5'-CGGGACAGTCTCCTTCTGAATGTA-3'	
P2Y_2_R sense	5'-CTGCCAGGCACCCGTGCTCTACTT-3'	340 bp
P2Y_2_R antisense	5'-CTGAGGTCAAGTGATCGGAAGGAG-3'	
P2Y_4_R sense	5'-GGCATTGTCAGACACCTTG-3'	530 bp
P2Y_4_R antisense	5'-AAGACAGTCAGCACCACAG-3'	
P2Y_6_R sense	5'-CGCTTCCTCTTCTATGCCA-3'	478 bp
P2Y_6_R antisense	5'-AGGCTGTCTTGGTGATGTG-3'	
MCP-1 sense	5'-CCTGTTGTTCACAGTTGCTGCC-3'	396 bp
MCP-1 antisense	5'-TCTACAGAAGTGCTTGAGGTGGTTG-3'	
IL-8 sense	5'-GAAGAT AGATTGCACCGATG-3'	365 bp
IL-8 antisense	5'-CATAGCCTCTCACACATTTC-3'	
GAPDH sense	5'-TCCCTCAAGATTGTCAGCAA-3'	309 bp
GAPDH antisense	5'-AGATCCACAACGGATACATT-3'	

### ELISA

C6 cells were plated using serum-free media in a 24-well plate at 3 × 10^5 ^cells/well and were allowed to acclimate for 24 h before starting the experiment. ATP was applied to the cells with or without gadolinium for 48 h and culture media were collected and analyzed with rat MCP-1 and rat IL-8 (GRO/KC) ELISA kit (Peprotech, Rockey Hill, NJ) according to the manufacturer's protocol. The detection limits were 47 and 16 pg/ml for MCP-1 and IL-8, respectively.

### Statistical analysis

Data are presented as means ± standard error of mean (SEM). Statistical significance (*p *< 0.05) was evaluated using one-way analysis of variance followed by Student-Newman-Keuls multiple comparison test (GraphPad Prism 3.0; San Diego, CA).

## Results

### ATP-induced changes in Ca^2+ ^mobilization

**Figure 1:**
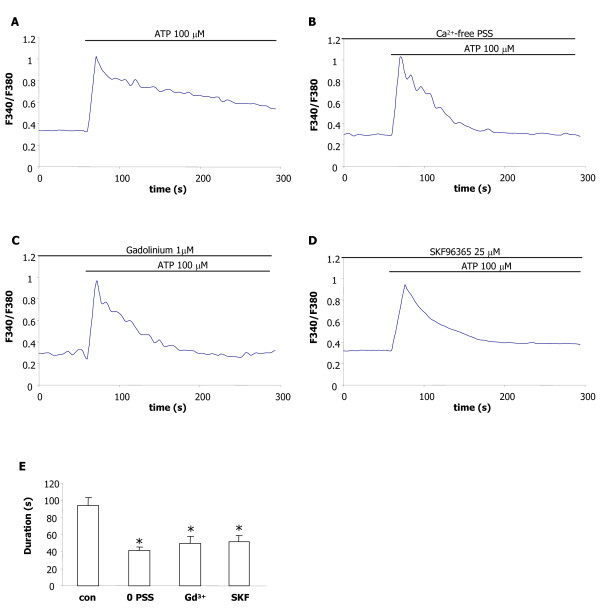
**ATP-induced changes in [Ca^2+^]i in C6 glioma**. (A) Representative response for ATP (100 μM) showing rapid and slow components of decay. (B) Typical ATP response in Ca^2+^-free PSS. (C) ATP response following gadolinium (Gd^3+^) pretreatment (1 μM, for 3 min). (D) ATP response in the presence of SKF96365 (25 μM, pretreatment for 3 min). (E) Quantification of durations of ATP responses for the different treatments (measured at one-half of peak response; N = 4/treatment). Values are mean ± SEM. *p < 0.001 compared with control.

Application of ATP (100 μM) to C6 glioma cells resulted in an early phase of increased [Ca^2+^]i which rapidly declined and was followed by a sustained component of response (Fig. 1A). In the presence of Ca^2+^-free PSS (0 PSS), ATP administration led to a single phase of [Ca^2+^]i mobilization representing only the rapid portion of the response (Fig. 1B). These results suggested ATP binding to a P2YR activating an inositol 1,4,5-trisphosphate (IP_3_)-mediated intracellular release of Ca^2+ ^and a subsequent entry of divalent ion through SOC [21,22]. To test this possibility two inhibitors of SOC, gadolinium and SKF96365, were applied prior to ATP stimulation. The concentrations of gadolinium (at 1 μM) [18] and SKF96365 (at 25 μM) [23,24] were chosen according to ones used in previous studies which were found effective for SOC inhibition. Representative ATP responses are presented with pre-treatment (3 min) with gadolinium (Fig. 1C) or SKF96365 (Fig. 1D). Exposure of C6 to either SOC inhibitor yielded a pattern of ATP response close to that observed in Ca^2+^-free PSS (Fig. 1B) with attenuation of the prolonged component of [Ca^2+^]i. Peak amplitudes of [Ca^2+^]i responses did not appreciably differ between control or the different treatments. Overall results are summarized in Fig. 1E (N = 4/treatment) and show durations (measured at half-maximal of peak value) of ATP responses were similar between Ca^2+^-free PSS and treatments with the two SOC inhibitors. Respective inhibitions of response durations relative to control ATP response were 44% (0 PSS), 49% (gadolinium) and 55% (SKF96365).

### Expression of purinergic P2YR in C6 glioma

**Figure 2:**
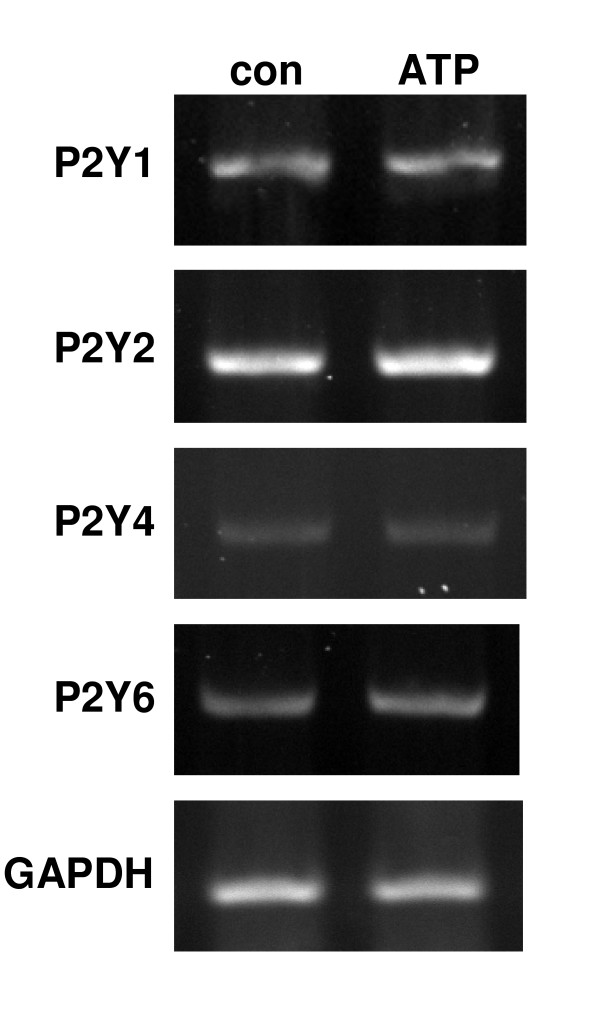
**Expression of P2YR linked to calcium stores in rodent C6 glioma**. The levels of receptor subtype P2Y_1_, P2Y_2_, P2Y_4 _and P2Y_6 _were examined in control and ATP-stimulated C6 cells (100 μM; 4 h exposure). The number of PCR cycles was 35 for all receptor subtypes. GAPDH served as a reaction standard. Data are representatives from 4 independent experiments.

RT-PCR experiments were undertaken to determine the expression of P2YR in unstimulated and ATP-stimulated C6 cells. The study examined only those P2YR known to be G protein coupled to IP_3_-dependent intracellular stores [21,22] and included P2Y_1_, P2Y_2_, P2Y_4 _and P2Y_6 _receptors. Typical expression of these subtype receptors are presented in Fig. 2 for control and ATP stimulation of C6 glioma for 4 h. Expression of mRNA for any of the subtype P2YR were unchanged between control and cells exposed to ATP treatment. The levels of band intensities occurred in the following order: P2Y_2_R > P2Y_1_R > P2Y_6_R > P2Y_4_R. Consistent results were obtained in three additional experiments with the intensity of P2Y_2_R exceeding levels for the other subtype P2YR for both control and ATP-stimulated glioma. Exposure of cells to longer-term ATP solution (48 h) had no significant effect to alter expression of any of the subtype P2YR (data not shown).

### Expression of chemokines MCP-1 and IL-8 in ATP-stimulated C6 cells

**Figure 3:**
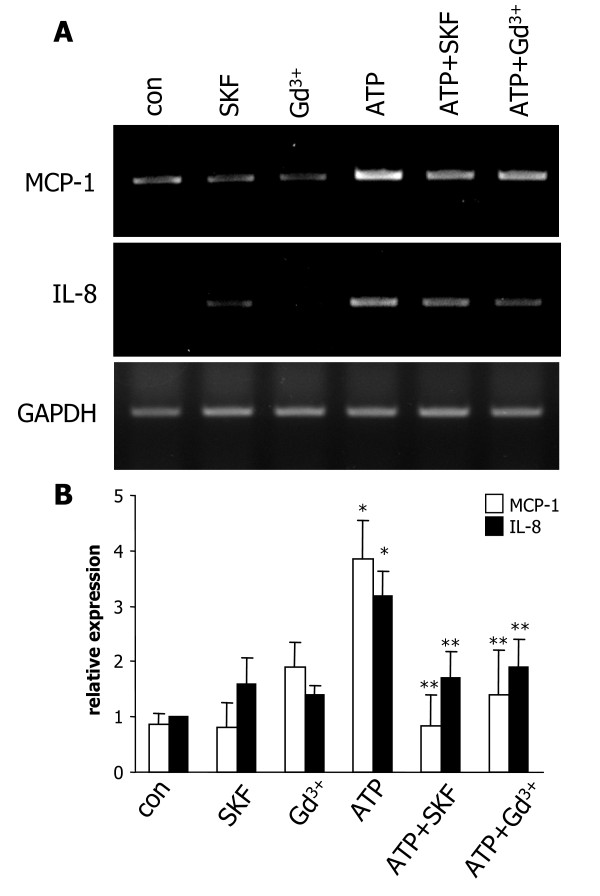
**Expression of MCP-1 and IL-8 in C6 cells**. (A) Representative levels for MCP-1 (upper row) and IL-8 (middle row) for the different treatments (4 h) applied to C6 glioma. Concentrations of ATP, SKF96365 and gadolinium (Gd^3+^) were the same as used in Ca^2+ ^studies (Fig. 1). GAPDH served as a reaction standard (lower row). (B) Overall results (N = 4 experiments) showing relative chemokine expression normalized with GAPDH. Values are mean ± SEM. *p < 0.05 compared with control, **p < 0.05 compared with ATP-treated group.

Chemokines, such as MCP-1 and IL-8, released from ATP-stimulated glioma could act to mobilize immune cells nearby tumor microenvironments. Representative RT-PCR for these two chemokines is presented in Fig. 3A for the different treatments (4 h exposure) applied to C6 glioma cells. Application of ATP (100 μM) markedly enhanced expression of MCP-1 and IL-8 relative to control. Treatments of SKF96365 (25 μM) or gadolinium (1 μM) with ATP attenuated chemokine expression compared with ATP alone. Application of SKF96365 or gadolinium alone showed mRNA for MCP-1 and IL-8 similar to control. Quantification for the relative expression of the two chemokines with the different treatments (N = 4/treatment) of C6 glioma is presented in Fig. 3B. ATP stimulation increased expression of MCP-1 by 4.4-fold and IL-8 by 3.2-fold relative to control. Expression of MCP-1 was significantly reduced with SKF96365 (by 78%) and gadolinium (by 64%) treatment of ATP-stimulated C6 relative to ATP application alone. The corresponding decreases for IL-8 were 46% with SKF96365 and 40% with gadolinium with both values representing significant effects of the SOC antagonists.

### Production of MCP-1 and IL-8 in ATP-stimulated C6 cells

**Figure 4:**
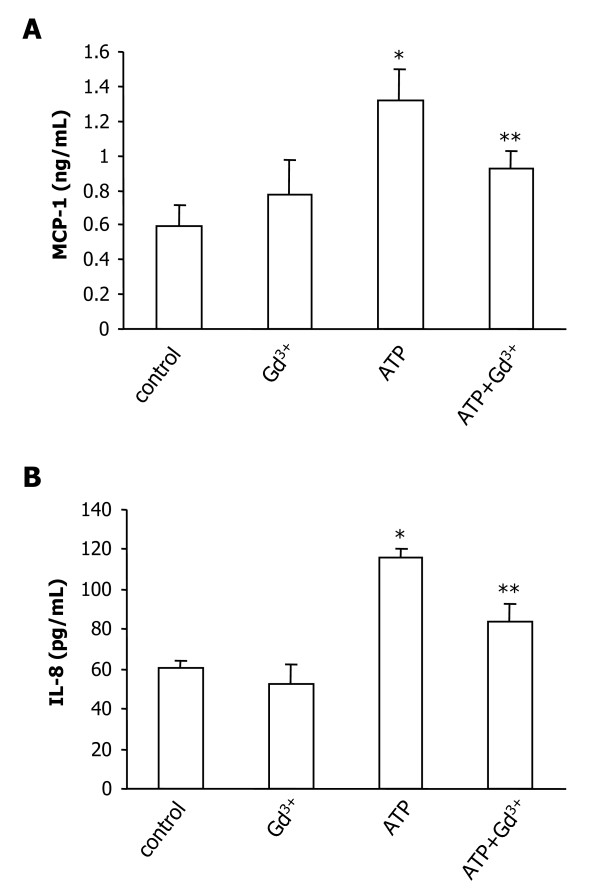
**Production of MCP-1 and IL-8 in C6 glioma**. C6 cells were treated with ATP (100 μM) in the presence, or absence, of gadolinium (Gd^3+^, 1 μM) for 48 h. (A) Levels of MCP-1 production (ng/ml) and (B) levels of IL-8 production (pg/ml) for the different treatments. Data are presented as mean ± SEM from 4 independent experiments. * indicates significant difference from control (*p *< 0.001) and ** indicates significant difference from ATP-treated group (*p *< 0.05).

We next examined if the enhancing effect of ATP and the inhibitory effect of SOC inhibitors on chemokine expression were translated at the protein levels. Preliminary ELISA studies showed that production of both MCP-1 and IL-8 was detectable as early as 4 h but in the low range of detection limits for up to 24 h after ATP stimulation. Subsequent experiments used a 48 h exposure of C6 glioma to ATP with gadolinium studied as an SOC inhibitor. Sustained exposure of C6 cells to SKF96365 (25 μM) caused cytotoxicity to cells thus precluding use of this compound for longer-term treatment. The overall results (N = 4/treatment) show MCP-1 production was increased by 131% with ATP stimulation compared with control (Fig. 4A). Inclusion of gadolinium with ATP significantly attenuated MCP-1 (by 64%) relative to ATP alone. Levels of IL-8 were also enhanced by ATP (by 92%) compared to control (Fig. 4B). Addition of gadolinium with ATP was effective in reducing levels of IL-8 by 54% compared to ATP applied alone.

## Discussion

The results from this work demonstrate that ATP stimulation of C6 glioma causes an increased cellular expression and production of the chemokines, MCP-1 and IL-8. These chemokines could serve as potent factors to mobilize tumor cells and also recruit microglia to help promote tumor development. Both expression and release of chemokines were inhibited by antagonists for SOC-mediated influx of Ca^2+ ^suggesting ATP induced the activation of a SOC-dependent pathway. Our findings lead to the novel suggestion that ATP binding to a metabotropic P2YR, coupled to activation of SOC, as an underlying mechanism for chemokine production in tumor cells. As discussed below, these findings are distinct from previous work reporting increased levels of these chemokines mediated by activation of the ionotropic P2X_7_R with the ligand, BzATP [16].

Calcium-sensitive spectrofluorometry showed ATP-induced increases in [Ca^2+^]i consisted of two components of response decay with the rapid and slow phases representing respective store release and plasmalemmal entry of Ca^2+^. In the presence of inhibitors of SOC, SKF96365 or gadolinium, the secondary but not initial, component of response was suppressed. Both SOC inhibitors were highly effective in blocking Ca^2+ ^entry since durations of ATP responses with either compound were similar to those measured using Ca^2+ ^free solution. It should be noted that no specific SOC antagonist has yet been identified with both SKF96365 and gadolinium capable of modulating other Ca^2+ ^entry pathways. The findings and implication for two Ca^2+ ^influx channels in glioma, SOC (this work) and P2X_7_R [16], are considered in more detail below.

RT-PCR was used to determine expression of metabotropic purinergic receptors P2Y_1_R, P2Y_2_R, P2Y_4_R and P2Y_6_R which are linked to IP_3_-mediated mobilization of [Ca^2+^]i [21]. Unstimulated C6 glioma showed a prominent band intensity for P2Y_2_R with a relatively lower basal expression for the other P2YR. The expression for any of the subtype P2YR was not altered with exposure of C6 cells to ATP (4 h treatment). Interestingly, longer-term exposure (48 h) of C6 glioma to ATP had no appreciable effect to alter mRNA expression for any of the P2YR (data not shown). Although our results show prominent expression of P2Y_2_R in glioma, future studies on protein levels and use of specific purinergic agonists and antagonists will have utility in linking specific P2YR with Ca^2+^-dependent responses. It is noteworthy that P2Y subtype receptors exhibit differential activation with purines with P2Y_1_R and P2Y_6_R more sensitive to ADP while P2Y_2_R and P2Y_4_R are more sensitive to ATP [21].

ATP stimulation of C6 glioma (4 h exposure) enhanced expression of the potent microglial-mobilizing chemokines, MCP-1 and IL-8. Expression of both chemokines were significantly reduced with SKF96365 or gadolinium included with the ATP stimulation. ELISA assay demonstrated that production of both chemokines was relatively low for ATP stimulation of C6 cells for durations less than 24 h but was enhanced with longer-term (48 h) exposure. In the latter case, levels of MCP-1 and IL-8 were significantly diminished with gadolinium included in the ATP treatment. It is noteworthy that the stimulatory effects of ATP on MCP-1 and IL-8 production were markedly lower compared to other agents releasing chemokines from astrocytic cell types. These agents include the P2X_7_R agonist, BzATP [25] and the pro-inflammatory cytokines TNF-α, and IL-1β [26,27].

The increased chemokine expression and production was not directly attributable to changes in receptor expression since none of the P2YR showed changes in band intensity with ATP treatment (Fig. 2). It is possible that the high basal levels of subtype P2Y_2_R expression in control would preclude RT-PCR analysis showing any increases in this subtype receptor after exposure of C6 cells to ATP. Gadolinium treatment was demonstrated to significantly reduce levels of MCP-1 and IL-8 in ATP-stimulated C6 cells. However, the inclusion of gadolinium with ATP did not diminish chemokine productions to control levels. Although P2X_7_R could contribute, this possibility is unlikely since ATP levels near 1 mM are generally required for activation of this receptor [28,29]. Our results do not exclude involvement of other P2XR since it is known that subtype receptors from the P2X family mediate influx of Ca^2+ ^into stimulated cells [12].

Together with previous data [16], C6 glioma cells are shown to express at least two different purinergic-dependent Ca^2+ ^entry pathways which modulate cellular releases of the chemokines, MCP-1 and IL-8. Activation of SOC subsequent to ATP binding to a P2YR or activation of P2X_7_R [16] with BzATP (ATP was not studied as an agonist) is coupled to C6 chemokine production. The overall results suggest that in C6 glioma, two different calcium influx pathways converge with the same functional endpoint of enhanced chemokine release. The effects of SOC inhibitors on rapid Ca^2+ ^responses and on longer-term cell functional responses indicates the involvement of Ca^2+^-dependent transcription factors in mediating tumor cell production of chemokines. One possibility is that release of chemokines such as MCP-1 and IL-8 from C6 are modulated by SOC activation with physiological ATP concentrations whereas the purinergic P2X_7_R pathway only contributes under extremely elevated levels of ATP.

Our findings suggest that pharmacological antagonism of SOC could serve as a rationale strategy to inhibit tumor growth. The SOC-dependent secretion of MCP-1 and IL-8 from C6 glioma would increase tumor cell mobility and also act as mobilization and proliferation signals to direct resident microglia into expanding tumors. It has been pointed out that recruitment of microglia could aid in supporting an immunosuppressive tumor environment to counter effects of T-cell-mediated killing of tumor cells [4]. However, recent findings also indicate that under certain conditions activated microglia/macrophages are capable of killing glioma cells [30,31]. Thus, it seems likely that microglia/macrophages could play both pro- or anti-neoplastic roles depending on the signaling factors present in the tumor microenvironment.

## Conclusions

Our results suggest ATP-induced Ca^2+ ^entry, mediated by activation of SOC in C6 glioma, as a mechanism linked to the cellular expression and release of chemokines. Increased levels of MCP-1 and IL-8 are predicted to enhance the mobility of tumor cells and promote recruitment of microglia into developing tumors thereby supporting tumor growth. Pharmacological inhibition of SOC in astroglioma is suggested as a novel treatment strategy to inhibit tumor progression.

## Competing interests

The authors declare that they have no competing interests.

## Authors' contributions

NJ designed research, performed research, analyzed data, and wrote the manuscript. HBC analyzed data JGM designed research, analyzed data, and wrote the manuscript. All authors have read and approved the final manuscript.

## Pre-publication history

The pre-publication history for this paper can be accessed here:

http://www.biomedcentral.com/1471-2407/9/442/prepub
